# The public image of nursing during the COVID‐19 pandemic: A cross‐sectional study

**DOI:** 10.1111/inr.12922

**Published:** 2023-12-05

**Authors:** Gamze Yavaş, Ayla Nur Özerli

**Affiliations:** ^1^ Department of Obstetrics and Gynecology Nursing Akdeniz University Kumluca Faculty of Health Sciences Antalya Turkey; ^2^ Nursing Department Student Akdeniz University Kumluca Faculty of Health Sciences Antalya Turkey

**Keywords:** COVID‐19, image, nursing, perception, public

## Abstract

**Objective:**

This study was conducted to assess the public image of the nursing profession during the COVID‐19 pandemic.

**Background:**

Before the COVID‐19 pandemic, the public was not as aware of the significance and indispensability of nursing. However, with the pandemic, the importance of nursing has gained prominence on national and international platforms.

**Methods:**

This was a cross‐sectional study. The study sample consisted of participants aged 18 and over who were Turkish speakers from seven regions of Turkey. Data were collected between May and July 2021 using an online survey, Image Scale for the Nursing Profession (ISNP), and Personal Information Form.

**Results:**

Five hundred and two individuals participated in the study. The average ISNP scores of the participants were 150.09 ± 9.62. It was found that participants who had a healthcare‐related occupation and were satisfied with the nursing care they received in the hospital had statistically higher ISNP total scores (*p* = 0.006, *p* = 0.000), respectively. The study revealed that the value, respect, and trust given to nurses increased with the COVID‐19 pandemic.

**Conclusion:**

The results suggest a notable enhancement in the public's view of the nursing profession amid the COVID‐19 pandemic.

**Implications for nursing and health policies:**

Developing promotional strategies for the nursing profession is crucial to enhance the public perception of the nursing profession. These strategies can help the public better understand and appreciate the nursing profession and the role of nurses in society. This requires the support of nurses, nursing educators, nurse managers, and policymakers.

## INTRODUCTION

Although the nursing profession has rapidly evolved in recent years, its public image has not developed at the same pace (Maliheh et al., 2020; Zamanzadeh et al., 2022). Since Florence Nightingale's time, the public image of nursing has been one of the main areas of struggle for the nursing profession (Moore et al., 2019). Nursing is perceived as a profession that is trusted but not sufficiently respected in society, with roles that are not fully understood, competence, autonomy, and independence not recognized, relatively unknown, and often invisible (Girvin et al., 2016; López‐Verdugo et al., 2021). The concept of nursing image is complex, inclusive, dynamic, and multidimensional (Girvin et al., 2016; Rezaei‐Adaryani et al., 2012). The nursing image consists of the nursing image in the media, nurses’ self‐image, public perception of nursing, and nurses’ perception of the public's image (Rezaei‐Adaryani et al., 2012).

Nursing's public image encompasses the collective beliefs, perceptions, and observations of individuals about nurses and the nursing profession (Morris‐Thompson et al., 2011). The public image of nursing is an important issue to address as it can influence an individual's decisions to select nursing as a profession, patient satisfaction and care quality, nurses’ motivation and job performance, quality of work life, intention to leave the profession, and health policies (Blau et al., 2023; Elmorshedy et al., 2020; Grinberg & Sela et al., 2022; Maliheh et al., 2020; Masih & Gulzar, 2018; Shen et al., 2022; Takase et al., 2006). Roshangar et al. (2021) stated that improving the professional image of nurses in the community can improve the quality of nurses' work life. A study conducted in Israel revealed that as nurses’ positive perceptions of nursing increased, their perceptions of the quality of care they provided also increased (Grinberg & Sela et al., 2022). A study in Vietnam also found a direct relationship between the public image of nursing and the quality of nursing care (Ha et al., 2016). In this context, it is extremely important to conduct studies evaluating the public perception of nursing.

Various factors such as mass communication tools, culture, stereotypes, and nurses’ self‐image perception affect the public image of nursing (González et al., 2023; Price et al., 2019). One of the factors that affect the public image of nursing is the role of nurses in disasters such as tsunamis, earthquakes, and outbreaks like the Middle East Respiratory Syndrome Coronavirus (MERS‐CoV). Such disasters create unique opportunities for the recognition of the values and true position of nursing in society (Taghinezhad et al., 2020; Zamanzadeh et al., 2022). Before the COVID‐19 pandemic, society was not as aware of the importance and indispensability of the nursing profession. However, with the pandemic, nurses have become a fundamental component in combating the outbreak and have played a crucial role in meeting the healthcare needs of the community. The pandemic has led to greater recognition of the importance of nursing due to the dedicated efforts of nurses (Lotfi et al., 2021; Padilha et al., 2020; Zhang et al., 2021). During the pandemic, the community showed significant appreciation, gratitude, and love toward nurses. The pandemic not only highlighted the strength of nursing but also brought forth certain realities and challenges faced by the nursing workforce (Bennett et al., 2020). In the days of the pandemic when everyone was distancing themselves from each other and avoiding physical contact, nurses became the “closest individuals” to patients. In 2020, the World Health Organization announced it as the “Year of the Nurse and Midwife.” The International Council of Nurses (2021) chose the theme for 2021 as “Nurses: A Voice to Lead—A Vision for Future Healthcare.” During the COVID‐19 pandemic, nurses performed their professional duties under increasingly challenging conditions and continued to provide nursing care. Nurses assumed caregiving, counseling, educational, leadership, and research roles at the highest levels during the pandemic (Chen et al., 2020; Choi et al., 2020; Smith et al., 2020). Although the healthcare and care needs of the community during the COVID‐19 outbreak heightened the importance of nursing, there have been limited studies examining the change in the perception of nursing in society.

### Aim

This study was conducted to assess the public image of the nursing profession during the COVID‐19 pandemic. The research questions for the study are as follows:

- What is the level of the public perception of nursing during the COVID‐19 pandemic?
- What are the factors that influence the public perception of nursing profession during the COVID‐19 pandemic?
- How has the public perception of nursing changed in conjunction with the COVID‐19 pandemic?

## METHODS

### Study design, study location, and study population

This was a cross‐sectional study and the sample consisted of participants over the age of 18 from seven regions of Turkey who were literate in Turkish. Data were gathered through the utilization of a “Personal Information Form” and the “Image Scale for the Nursing Profession” (ISNP).

### Study variables and data collection tools

#### Personal information form

The Personal Information Form included questions related to the participant's age, education, place of residence, family type, income, marital status, occupation, being in the presence of nurses working in the environment, previous hospitalization and companionship status, satisfaction with previous nursing care, and being diagnosed with COVID‐19.

#### Image scale for the nursing profession

Dost and Bahçecik (2015) developed the ISNP to assess nurses' perceptions of their professional image. The scale was used to find out the perception of the public toward the nursing profession in this study. The scale is composed of six sub‐dimensions, which cover professional qualifications, working conditions, gender, education, professional status, and appearance. The scale is a five‐point Likert‐type and consists of 42 items. The score obtained from the scale varies between 42 and 210, and a total score of 42–75 means “very weak,” 76–109 means “weak,” 110–143 means “moderate,” 144–177 means “good,” and 178–210 means “very good” image perceptions. As the score on the scale increases, the public perception of the nursing profession becomes increasingly positive. In the original scale development study conducted by Dost and Bahçecik (2015), the Cronbach alpha coefficient for the overall scale was reported as 0.88, whereas in our study, it was found to be 0.83.

#### Data collection

The study data were gathered online between May and July 2021. The data collection forms were prepared using Google Forms. The researchers shared with the participants the data collection tools from personal social networking sites such as Instagram, Twitter, Facebook, and WhatsApp. It was impossible to send the questionnaire incompletely, thanks to the Google Forms function that prevents sending partially answered or unanswered questions.

#### Data analysis

Descriptive statistics, one‐way ANOVA, independent sample *t* test, and Pearson correlation analyses were performed using the SPSS 24.0 program. In the study, a 95% confidence interval and a significance level of *p* < 0.05 were accepted.

#### Ethical considerations

The study adhered to the principles outlined in the Helsinki Declaration. To conduct this study, approval was obtained from the Turkish Ministry of Health (Research No: 2021‐04‐21T11_30_46) and the Akdeniz University Clinical Research Ethics Committee (No: 337, May 26, 2021). At the beginning of the data collection form, an informed consent text informed the participants about the research. After participants checked the consent box to indicate their willingness to participate in the study, they were able to respond to the data collection tool. The names of the participants were not collected in the study.

## RESULTS

In this part of the study, the data are presented as percentages, and the data of the majority of the participants are presented to avoid data duplication. Five hundred and two individuals participated in the study. The average age of the participants was 28.51 ± 9.85 (min: 18, max: 72), and 67.3% of them were between the ages of 18 and 28. 61.6% of the participants were women, 50.6% were university graduates, 68.1% lived in a provincial center, 77.3% had a nuclear family structure, 54.8% had an income equal to their expenses, and 74.7% were single. 71.7% of the participants indicated that they did not work in a health‐related profession, 60.8% had been in the presence of a nurse working around them, 62.7% had stayed in a hospital as a companion, 70.7% had received care from a nurse before, and 77.3% were satisfied with that nursing care. 18.7% of the participants expressed that they had been diagnosed with COVID‐19.

ISNP total and subscale mean scores and min–max values are given in Table 1. The mean score in the “professional qualifications” sub‐dimension of the ISNP was 45.38 ± 5.18, the mean score in the “working conditions” sub‐dimension was 27.61 ± 3.86, the mean score in the “gender” sub‐dimension was 27.19 ± 4.73, the mean score in the “education” sub‐dimension was 19.52 ± 2.25, the mean score in the “professional status” sub‐dimension was 19.43 ± 3.84, and the mean score in the “appearance” sub‐dimension was 10.94 ± 2.84. The participants’ average scores in the “working conditions” sub‐dimension were low, whereas those in the “gender” and “appearance” sub‐dimensions were moderate, and their average scores in the “professional qualifications,” “education,” and “professional status” sub‐dimensions were at a good level. When the lowest and highest scores obtainable from the overall scale (minimum: 42; maximum: 210) were compared with the mean scores obtained by the participants (150.09 ± 9.62), it could be seen that the participants’ image perception scores for the nursing profession were good (Table 1).

**TABLE 1:** The total and sub‐dimension scores of the ISNP

Item	Number of items	Min–Max	X ± SD	The score range of participants
Professional qualifications	11	11–55	45.38 ± 5.18	15.00–51.00
Working conditions	10	10–50	27.61 ± 3.86	17.00–50.00
Gender	8	8–40	27.19 ± 4.73	12.00–37.00
Education	5	5–25	19.52 ± 2.25	7.00–25.00
Professional status	5	5–25	19.43 ± 3.84	7.00–25.00
Appearance	3	3–15	10.94 ± 2.84	3.00–15.00
**Total**	42	42–210	150.09 ± 9.62	121.00–174.00

The comparison of the total mean scores obtained from the ISNP according to some descriptive characteristics of the participants is given in Table 2. It was found that there was no statistically significant difference in the total mean score for gender, education, place of residence for the longest time, family type, income, marital status, presence of nurses working in the environment, previous hospitalization, receiving care from a nurse before, staying as a companion before, and being diagnosed with COVID‐19 (*p* > 0.05). It was found that there was a statistically significant difference in the total mean score on the ISNP according to the participants’ status of working in a health‐related profession and being satisfied with previous nursing care (*p* = 0.006, *p* = 0000, respectively). Accordingly, it was found that the participants who worked in a profession related to health and who were satisfied with the nursing care they had received before had a good image of the nursing profession. In addition, a weak and positive statistically significant relationship was found between the age of the participants and the mean score on the ISNP (*p* = 0.012, *r* = 0.112). In light of this, it can be concluded that as participants' age increased, their perceptions of the nursing profession became more favorable.

**TABLE 2:** ISNP total scores according to some characteristics of the participants

Variable	*n*	%	X ± SD	Test value	*p*
**Gender**					
Female	309	61.6	150.29 ± 9.61	*t* = 0.573	0.567
Male	193	38.4	149.78 ± 9.67		
**Educational status**					
Literate	13	2.6	150.75 ± 8.10	*F* = 0.464	0.803
Primary school graduate	11	2.2	152.50 ± 10.53		
Secondary school graduate	15	3.0	145.00 ± 14.82		
High school graduate	153	30.5	150.71 ± 9.31		
University graduate	254	50.6	149.67 ± 9.29		
Postgraduate	56	11.2	150.32 ± 10.55		
**Place of residence for the longest time**					
Provincial center	342	68.1	149.54 ± 9.56	*F* = 1.110	0.330
District	110	21.9	150.70 ± 8.72		
Village/town	50	10.0	151.77 ± 11.55		
**Family type**					
Nuclear	388	77.3	149.90 ± 9.53	*t* = −0.848	0.397
Extended	114	22.7	150.77 ± 9.95		
**Income status**					
Income is less than expenses	121	24.1	148.67 ± 9.47	*F* = 1.746	0.175
Income equals expenses	275	54.8	150.52 ± 9.56		
Income is greater than expenses	106	21.1	150.61 ± 9.90		
**Marital status**					
Married	127	25.3	151.53 ± 9.56	*t* = 1.950	0.052
Single	375	74.7	149.61 ± 9.61		
**Working in a health‐related profession**					
Yes	142	28.3	151.99 ± 9.41	*t* = 0.732	**0.006**
No	360	71.7	149.35 ± 9.62		
**Presence of nurses working in the environment**					
Present	305	60.8	150.19 ± 9.01	*t* = 0.253	0.800
Absent	197	39.2	149.95 ± 10.53		
**Receiving care from a nurse before**					
Received	355	70.7	150.61 ± 9.45	*t* = 1.855	0.064
Did not receive	147	29.3	148.86 ± 9.95		
**Staying in the hospital as a companion before**					
Stayed	315	62.7	149.67 ± 9.38	*t* = −1.289	0.198
Did not stay	187	37.3	150.81 ± 10.00		
**Satisfaction with previous nursing care**					
Satisfied	274	77.3	151.52 ± 9.39	*F* = 18.674	**0.000**
Partially satisfied	61	17.1	148.25 ± 9.16		
Not satisfied	20	5.6	139.50 ± 7.43		
**Receiving a diagnosis of COVID‐19**					
Diagnosed	94	18.7	149.54 ± 9.79	*t* = −0.622	0.534
Not diagnosed	408	81.3	150.22 ± 9.59		

The significance level of p<0.05 was considered in the study.

The perceptions of the participants toward the nursing profession during the COVID‐19 pandemic are presented in Table 3. 97.2% of the participants stated that the workload of nurses increased, and 95.2% stated that their working conditions became heavier due to the COVID‐19 pandemic process. 64.7% of the participants stated that the value given to nurses by society increased, 64.5% stated that the prestige of nurses increased, and 68.7% stated that the confidence in nurses increased due to the COVID‐19 pandemic process. In addition, 60.2% of the participants stated that the exposure of nurses to violence increased during the COVID‐19 pandemic.

**TABLE 3:** Participants’ perceptions of nursing in the COVID‐19 process

Variable	*n*	%
**Workload of nurses**		
Increased	488	97.2
Did not change	11	2.2
Decreased	3	0.6
**Working conditions of nurses**		
Became heavier	478	95.2
Did not change	21	4.2
Became easier	3	0.6
**The value that society places on nurses**		
Increased	325	64.7
Did not change	158	31.5
Decreased	19	3.8
**Respect for nurses**		
Increased	324	64.5
Did not change	156	31.1
Decreased	22	4.4
**Confidence in nurses**		
Increased	345	68.7
Did not change	145	28.9
Decreased	12	2.4
**Nurses’ exposure to violence**		
Increased	302	60.2
Did not change	144	28.7
Decreased	56	11.2

## DISCUSSION

This study contributes to the literature by examining the public's image of nursing during the COVID‐19 pandemic. In our study, it was found that participants had a good level of perception of nursing. In the literature, it has been shown that the public perception of nursing was poor or moderate before the COVID‐19 pandemic and that this perception did not change over the years (Baykara Mat & Baykal, 2021; Elmorshedy et al., 2020; Gul, 2008, 2008; Maliheh et al., 2020; ten Hoeve et al., 2014). On the other hand, studies have shown that the public perception of nursing has improved with the COVID‐19 pandemic (Blau et al., 2023; Kim & Lee, 2023; Yalçın et al., 2023; Yılmaz Güven & Şener, 2023). Rubbi et al. (2017) demonstrated that, in Italy, the post‐pandemic period witnessed a change in the public perception of nursing, with society acknowledging the irreplaceable competence and importance of nurses within the healthcare system. In summary, these findings indicate a significant improvement in the public perception of nursing during the COVID‐19 pandemic and an increased awareness of the role and importance of nurses. Especially at the onset of the pandemic, nurses’ dedication to providing care to patients, even under challenging working conditions such as risking their health, increased workload, long working hours, and a shortage of personal protective equipment, drew the attention of the community. This sacrifice contributed to a positive change in the public perception of nurses. To sustain and reinforce this positive perception of the nursing profession in the post‐pandemic period, it is recommended to organize awareness campaigns regarding the roles of nurses within healthcare systems. Additionally, conducting further research to monitor the sustainability of this positive image in the post‐pandemic period is suggested.

This study has demonstrated that participants with healthcare‐related professions have a positive perception of nursing. The study by Yılmaz Güven and Şener (2023) also supports our findings. Working in a healthcare‐related profession may have contributed to having more knowledge and experience regarding the healthcare sector and healthcare professionals, having the opportunity to work with nurses, and collaborating with them, all of which could lead to a more detailed understanding of the nursing profession and the formation of a positive image.

This study has demonstrated that participants engaged in healthcare‐related professions have a positive perception of nursing. Similar studies conducted during the COVID‐19 pandemic (Karakaplan & Ulupınar, 2023; Yılmaz Güven & Şener 2023, ) have also supported our findings. This indicates that positive patient experience and satisfaction can influence the image of nursing. Therefore, it emphasizes the importance of nurses focusing on communication, empathy, and the quality of care to improve patient satisfaction and create a positive professional image.

Our findings emphasize the importance of improving nurses' working conditions to enhance the public perception of nursing during the COVID‐19 pandemic. The fact that almost all of the participants stated that the workload of nurses increased and working conditions became heavier during the pandemic clearly shows the urgency of this issue. Furthermore, the fact that participants scored the “working conditions” sub‐dimension lowest in the ISNP supports this finding. Similarly, in Uysal and Demirdağ (2022), participants reported that nurses continued to provide care by putting their own lives at risk and that working conditions had become even more challenging compared with before the pandemic. These findings underscore the need for healthcare institutions and governments to review and improve nurses’ working conditions. Ensuring that nurses have safe working environments and manageable workloads can contribute to greater appreciation and respect for the nursing profession in society. Additionally, collaborating with the media and the public to emphasize the importance of nursing and draw attention to the challenges nurses face can play a significant role in this process.

This study demonstrates an increase in the value, respect, and trust given to nurses during the COVID‐19 pandemic. Similarly, other studies have also shown that the pandemic has increased appreciation for nurses (Arda Sürücü et al., 2023; Begnini et al., 2021; Foà et al., 2021; Karakaplan & Ulupınar, 2023; Şahan et al., 2021; Tokac et al., 2022; Zamanzadeh et al., 2021). In Blau et al. (2023), participants mentioned that the prestige of nursing increased in society with the pandemic, and awareness of the nursing profession and the nurse's role also increased. Uysal and Demirdağ (2022) noted that this positive perception of nurses in society may be temporary and could be forgotten after the pandemic. In a study comparing the pre‐pandemic and post‐pandemic periods, the finding of a decrease in society's trust and respect for health professionals after the pandemic is concerning (Stolzenberg et al., 2023). Nurses, nurse managers,nursed educators and professional associations and nursing educators should make efforts to sustain and reinforce this positive image. These efforts should aim to enhance public awareness of aspects such as the qualifications, education, and working conditions of nursing.

It is indeed concerning to demonstrate an increase in violence against nurses during the COVID‐19 pandemic. Although the sacrifices and efforts of health professionals during the pandemic are appreciated by society, studies showing an increase in violence underscore the presence of a serious issue (Arafa et al., 2021; Ghareeb et al., 2021). These findings emphasize the urgent need for action to prevent violence in the healthcare sector and ensure the safety of health professionals. In this context, healthcare institutions, governments, and civil society organizations should make greater efforts to prevent violence against health professionals and organize educational and awareness‐raising activities aimed at violence prevention. Additionally, awareness campaigns promoting an understanding of the importance and sensitivity of health professionals to the community and fostering an attitude against violence are crucial. Such initiatives can assist healthcare workers in providing their services in a safe and respectful working environment.

## CONCLUSION

This study demonstrates that the public perception of nursing is at a good level and that the COVID‐19 pandemic has positively influenced this perception. The study shows that some demographic factors did not affect this perception, but participants who work in healthcare‐related professions and are satisfied with nursing care have a more positive image. Additionally, alongside the positive view that the value, trust, and respect for nurses have increased during the pandemic, there are concerning findings regarding the increase in violence. This study emphasizes the necessity for further research and awareness campaigns to sustain and strengthen the positive public perception of nursing.

### Limitations

One of the strengths of this study is that it was conducted online, allowing participants from across the country to express their opinions. However, the reliance on self‐reporting by participants and the inability to reach individuals without smartphones or those who do not use social media are limitations of the research.

### Implications for nursing and health policy

The pandemic has presented a significant opportunity for the promotion of the nursing profession, influencing healthcare policies, and shaping future nursing practices. While combating pandemics like COVID‐19, it is evident that the vital roles of nurses in healthcare systems need to be better communicated to the public. Governments and nursing leaders should establish the necessary infrastructure to promote the nursing profession as an independent field that requires higher education and specialization. Nursing managers and nurses themselves can use online platforms and social media to introduce the nursing profession and its roles to the public, aiming to enhance public trust in nurses. Nursing organizations should plan projects to promote the nursing profession in the community, use print and visual media to showcase nurses' roles and contribute to projects such as films and documentaries that depict the experiences and efforts of nurses during pandemics. The pandemic may have contributed to a better public perception of nursing, but nurses have continued to provide services under challenging working conditions. Governments should improve healthcare service organizations, provide safer working conditions for nurses, enhance measures to prevent violence against healthcare workers, and make plans to better prepare for future health crises.

## AUTHOR CONTRIBUTIONS

GY: Study design. GY, AÖ: Data collection. GY, AÖ: Data analysis. GY: Study supervision. GY, AÖ: Manuscript writing. GY, AÖ: Critical revisions for important intellectual content.

## CONFLICT OF INTEREST STATEMENT

No potential conflict of interest was reported by the authors.

## FUNDING INFORMATION

This study did not receive any specific grant from funding agencies in the public, commercial, or not‐for‐profit sectors.

## ETHICAL STATEMENT

To conduct this study, approval was obtained from the Scientific Research Platform of the Ministry of Health of the Republic of Turkey (Research No: 2021‐04‐21T11_30_46) and the Akdeniz University Clinical Research Ethics Committee (No: 337, May 26, 2021).
